# Physical modalities inducing immunogenic tumor cell death for cancer immunotherapy

**DOI:** 10.4161/21624011.2014.968434

**Published:** 2015-01-07

**Authors:** Irena Adkins, Jitka Fucikova, Abhishek D Garg, Patrizia Agostinis, Radek Špíšek

**Affiliations:** 1 Sotio; Prague, Czech Republic; 2 Department of Immunology; 2nd Faculty of Medicine and University Hospital Motol; Charles University; Prague, Czech Republic; 3 Cell Death Research and Therapy (CDRT) Unit; Department of Molecular and Cell Biology; University of Leuven (KU Leuven); Leuven, Belgium

**Keywords:** cancer immunotherapy, high hydrostatic pressure, hyperthermia, immunogenic cell death, ionizing irradiation, photodynamic therapy with hypericin, ATP, Adenosine triphosphate, CRT, calreticulin, DAMPs, danger-associated molecular patterns, DC, dendritic cells, EGFR, endothelial growth factor receptor, eIF2α, eukaryotic translation initiation factor 2α, ER, endoplasmic reticulum, HHP, high hydrostatic pressure, HMGB1, high-mobility group box 1, HT, hyperthermia, ICD, immunogenic cell death, HSP, heat shock protein, Hyp-PDT, Hypericin-based Photodynamic therapy, IFNγ, interferon-γ, NDV, Newcastle Disease Virus, ROS, reactive oxygen species, RT, radiotherapy, TLR, Toll-like receptor, UVC, ultraviolet C light

## Abstract

The concept of immunogenic cancer cell death (ICD), as originally observed during the treatment with several chemotherapeutics or ionizing irradiation, has revolutionized the view on the development of new anticancer therapies. ICD is defined by endoplasmic reticulum (ER) stress response, reactive oxygen species (ROS) generation, emission of danger-associated molecular patterns and induction of antitumor immunity. Here we describe known and emerging cancer cell death-inducing physical modalities, such as ionizing irradiation, ultraviolet C light, Photodynamic Therapy (PDT) with Hypericin, high hydrostatic pressure (HHP) and hyperthermia (HT), which have been shown to elicit effective antitumor immunity. We discuss the evidence of ICD induced by these modalities in cancer patients together with their applicability in immunotherapeutic protocols and anticancer vaccine development.

## Introduction to Immunogenic Cell Death

The contribution of the immune system to the therapeutic outcome of cancer treatment regimens involving surgery, radiotherapy (RT) or chemotherapy has been mostly neglected as the development of new therapies had primarily focused on tumor-cell killing for a long time. Of note, only in the case of RT, there was some early circumstantial evidence of contribution of immune system toward positive therapeutic response in the form of “abscopal effects.”^1^ Reported for the first time in 1953,^2^ abscopal effect was described as a phenomenon wherein RT could reduce tumor growth at distant sites outside the field of radiation possibly through anticancer immunity.^1^ However, the immunological mechanism behind these abscopal effects and its reliability, or therapeutic reproducibility, remained controversial for a long time thereby impeding its establishment as a therapeutically-exploitable paradigm. Overtime though, it became evident that an antitumor immune response plays a major role in the therapeutic success of cancer treatment in general, and mediates long-term survival of experimental animals.^3-7^

Many chemotherapeutic agents exert their cytotoxic effects by the induction of tumor cell apoptosis which has been historically regarded as a non-inflammatory, immunologically silent or even tolerogenic mode of cell death.^8^ This was challenged by a series of observations made more than a decade ago which showed that DCs can engulf apoptotic tumor cells and cross-present internalized antigens on MHC class I molecules to CD8^+^ T cells.^9^ Apoptotic tumor cells were also shown to elicit an effective antitumor immune response in mice.^10^ More recently, owing to a series of different studies, two morphologically equivalent but immunologically distinct subcategories of apoptosis, i.e., immunogenic and non-immunogenic apoptosis, were described giving rise to the new concept of immunogenic cell death (ICD).^4,5^ Dying the immunogenic way is, however, not unique to apoptosis. Cells dying by other cell death pathways such as necrosis/necroptosis or pyroptosis also induce cell death associated with immunogenicity.^11,12,13^ In fact, immunogenicity and inflammation associated with necrosis/necroptosis or pyroptosis was discovered before apoptotic ICD was characterized. In spite of this, it is not yet completely clear to what extent the molecular nature of the danger signals, which are mainly passively exposed by necrotic dying cancer cells, overlaps with that of immunogenic apoptosis. In this respect, it is even less clear whether during caspase-1 driven pyroptosis, a cell death pathway reported to occur predominantly in bacterially infected macrophages and dendritic cells, results in the release of similar immunogenic signals. Thus in this review we will focus on the mediators of immunogenic apoptosis.

**Figure 1.**
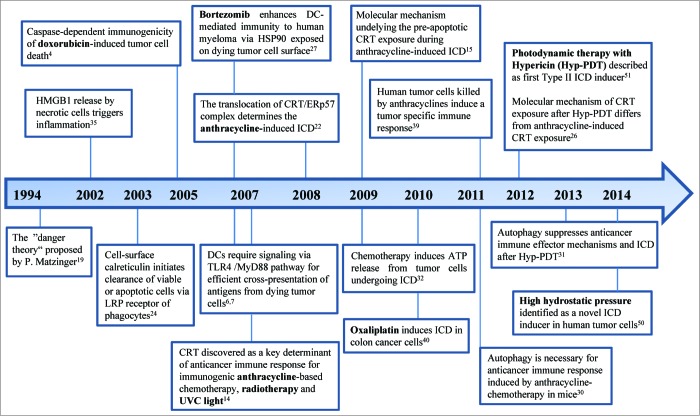
Timeline of the most important discoveries in the field of immunogenic cell death. Abbreviations: ATP, Adenosine triphosphate; CRT, calreticulin; DC, dendritic cell; HMGB1, high mobility group box 1; HSP90, heat shock protein 90; Hyp-PDT, photodynamic therapy with hypericin; ICD, immunogenic cell death; LRP, lipoprotein receptor-related proteins; MyD88, myeloid differentiation primary response gene 88; TLR, toll like receptor; UVC, ultraviolet light C.

The first immunogenic inducer doxorubicin, which belongs to the anthracycline family, was identified in 2005 by the group of Guido Kroemer and Laurence Zitvogel.^4^ The subsequent important discoveries in the field of ICD are shown in a timeline in **Fig. 1**. Later, it was found that murine tumor cells treated not only with doxorubicin, but also with mitoxantrone, oxaliplatin or ionizing irradiation underwent ICD and elicited strong anticancer immune responses in mice in the absence of any adjuvants. The authors also showed that the capacity of dying tumor cells to generate immune response is dependent on the cell death – inducing stimulus as tumor cells treated with some other cytotoxic agents like mitomycin C, cisplatin, thapsigargin or etoposide failed to induce ICD.^4,5,14^

Several conditions must be fulfilled in order to define tumor cell death as immunogenic. ICD has been found to depend on the concomitant generation of ROS and activation of ER stress (either resulting from or accentuated by this ROS production).^15-17^ Stressed tumor cells undergoing ICD start to expose on their cell surface, and release or secrete into their vicinity, a variety of damage-associated molecular patterns (DAMPs)^18,19^ which under physiological conditions have mostly non-immunological functions inside the cell. Only when exposed or emitted through complex and elaborate danger signaling trafficking module^20^ these molecules act as danger signals thereby determining the immunogenicity of a dying tumor cell in a context-dependent fashion.^21^ The list of DAMPs crucial for ICD includes (1) the pre-apoptotic surface exposure of calreticulin (CRT),^5,22-24^ (2) the pre-apoptotic or blebbing stage-associated secretion of adenosine triphosphate (ATP),^25,26^ (3) surface-exposure of heat shock protein 70 and 90 (HSP70 and HSP90)^27^ and (4) the release of high mobility group box 1 (HMGB1) or other toll-like receptor (TLR) agonists like HSP70.^6,28^ Moreover, it seems that not only the amount or diversity of immunogenic signals but also the defined spatiotemporal pattern of their emission determines the immunogenicity of dying tumor cells.^29^ Interestingly, autophagy was shown to be indispensable for anticancer immune response induced by anthracycline-chemotherapy in mice.^30^ On the other hand, the induction of autophagy in dying cancer cells suppressed anticancer immune effector mechanisms and ICD after photodynamic therapy with hypericin (Hyp-PDT).^31^

The cell surface exposure of CRT or the release ATP from dying tumor cells during ICD seems to be an active process and involves the participation of several intracellular proteins.^15,22,26,32^ Interestingly, the molecular signaling pathways which lead to CRT exposure or ATP release seem to involve an overlapping and also unique set of signaling proteins depending on the ICD inducer.^33,34^ The release of other DAMPs such as HMGB1 seems to be rather a passive event due to the disintegration of plasma membrane of the dying tumor cells.^29,35^ However, little is known about the intracellular mechanism of cell surface exposure or release of other danger molecules such as HSP70/90 proteins in cancer cells undergoing ICD. The immunogenic DAMPs bind to respective immune receptors e.g. pattern recognition receptors (PRRs) (TLRs for HMGB1/HSP70), phagocytosis or scavenger receptors (CD91 for surface exposed CRT/HSP90) and purinergic receptors such as P_2_X_7_R or P_2_Y_2_R (for ATP). This leads to the recruitment of innate immune cells to the tumor bed.^33,36^ The interaction of DAMPs with their cognate immune receptors facilitates the engulfment of tumor antigens and their cross-presentation to T cells. These processes lead to a potent IL-1β- and IL-17-dependent, IFNγ-mediated immune response involving γδ T cells/cytotoxic αβ T lymphocytes and tumor eradication.^36^ Interestingly, it has been reported that γδ T cells, in contrast to αβ T cells, may themselves possess TLRs.^37^ However, it has not yet been analyzed whether the ICD-associated TLR-binding of DAMPs might be directly activating γδ T cells thereby partly bypassing the DC-T cell interaction route. This represents an attractive possibility that needs further analysis.

From this point of view, cancer cell death can be further defined as immunogenic provided that the tumor-rejecting immunity is elicited in mice after immunization with syngeneic dying tumor cells in the absence of any adjuvant. Thus, ICD inducer must exert, at least in part, the therapeutic efficacy *in vivo* leading to a reduction or eradication of the tumor mass.^36^

The growing list of the ICD inducers, exhibiting all the major checkpoints determining the immunogenicity of cell death as described above, have been recently divided into two groups. These groups are based on their ability to trigger both cancer cell death as well as danger signaling as a consequence of direct induction of ER-stress (Type II inducers), or whether the inducer evokes ER stress-based danger signaling and apoptosis/cell death through convergent, but mechanistically separate targets (Type I inducers).^33,38^ Type I inducers of ICD such as anthracyclines,^4,39^ oxaliplatin,^40^ shikonin,^41^ 7A7 (murine EGFR-specific antibody),^42^ cyclophosphamide,^43^ bortezomib,^27^ cardiac glycosides,^44^ septacidin,^45^ bleomycin,^46^ ultraviolet C light (UVC),^14^ wogonin,^47^ vorinostat,^48^ γ-irradiation^14^ and newly described HHP^49,50^ target mainly cytosolic proteins, plasma membrane channels or proteins, or DNA replication and repair machinery, rather than primarily targeting the ER.^33^ On the other hand, Type II inducers which specifically target the ER include PDT with Hypericin (Hyp-PDT),^51^ and various different oncolytic viruses. Oncolytic viruses such as adenovirus, coxsackievirus B3,^33,38^ measles virus, vaccinia viruses, herpes simplex virus or Newcastle disease virus^13^ have been shown to induce various modes of ICD,^11^ however, the underlying molecular mechanisms remains to be determined. Of note, the Newcastle disease virus is the only oncolytic virus shown so far to induce both ICD^13^ as well as “abscopal effect”-like antitumor immunity as the localized intratumoral therapy with Newcastle disease virus leads to lymphocyte infiltration and antitumor effect in distant tumors without direct contact between the latter tumors and this virus.^52^ In **Table 1**, we summarize scarce data available on the induction of anticancer immunity in patients by Type I and Type II inducers as evidenced by ICD determinants or by eliciting tumor-antigen specific T cell responses. More clinical trials showing the impact of immunogenicity on the prognosis of cancer patients are awaited.

**Table 1.** The evidence of immunogenic cell death induction by Type I and Type II in cancer

ICD inducer	Cellular target for cell death induction	Evidence of antitumor immunity in patients connected to ICD determinants
**Type I**		
Anthracyclines, Mitoxantrone	DNA or proteins of DNA replication machinery	Breast cancer patients bearing a wt P2RX7 allele^25^ or a wt TLR4 allele^7^ benefited more from anthracycline therapy in comparison to those bearing mutated allele.
7A7 (EGFR-specific antibody)	EGFR	ND
Bortezomib	ERAD, 26S proteasome, CIP2A	Bortezomib improves progression-free survival in multiple myeloma patients overexpressing PRAME antigen^124^
Cardiac glycosides (CGs)*	Na^+^, K^+^-ATPase in plasma membrane	Retrospectively, a positive impact of administration of the cardiac glycosides digoxin during chemotherapy on overall survival in cohorts of breast, colorectal, head and neck, and hepatocellular carcinoma patients has been shown^44^
Cyclophosphamide ^†^	DNA	CTX induced a slight decrease of Tregs in the blood of patients with metastatic carcinoma treated with CTX (in combinantion with BCG injected in metastasis)^125^
CTX induced drop in B-cell counts, without affecting the number of T cells in cancer patients^126^		
Oxaliplatin	DNA synthesis	Patients bearing loss-of-function allele of TLR4 showed shorter progression-free survival and overall survival in comparison with patients bearing WT allele of the TLR4^40^
Shikonin	Tumor-specific pyruvate kinase-M2 protein, 20S subunit of proteasome	Initiation of clinical study (breast cancer NCT01287468)
UVC irradiation	DNA	ND
γ-irradiation	DNA	Better survival of patients with esophageal squamous cell carcinoma (ESCC) after radiotherapy and chemotherapy treatment (increased HMBG1 in serum)^57^
Septacidin	Cellular Proteins possibly Nrf2 and Tyrosyl-DNA phosphodiesterase	ND
Bleomycin^‡^	DNA	ND
High hydrostatic pressure	Cellular proteins	ND
Wogonin	Mitochondria	ND
Vorinostat (histone deacetylase inhibitor)	Histones (Nucleus)	ND
**Type II**		
Hypericin-based Photodynamic therapy	Endoplasmic reticulum	ND
Various Oncolytic Viruses	Endoplasmic reticulum	ND

*It is important to note that CGs alone are unable to induce ICD *in vivo*; for that to happen they need to be combined with other chemotherapeutics although those can be non-ICD inducers whose immunogenicity can be reinstated by CGs.

^†^ Immunopotentiating effects of cyclophosphamide are highly dose dependent in both humans as well as preclinical animal models. Metronomic doses of cyclophoshamide have been found to be “ICD-supportive” however high doses can be strongly immunosuppressive.

^‡^ Bleomycin has been shown to exert ambivalent immune effects since along with ICD induction it paradoxically also induces proliferation of immunosuppressive Treg cells.

Abbreviations: CG, cardiac glycosides; CIP2A, cancerous inhibitor of PP2A; CTX, cyclophosphamide; EGFR, epidermal growth factor receptor; ESCC, esophageal squamous cell carcinoma; ERAD, endoplasmic-reticulum-associated protein degradation; HMGB1, high mobility group box 1; ICD, immunogenic cell death; ND, not defined; NDV, Newcastle disease virus; Nrf2, nuclear factor erythroid 2-related factor; PRAME, preferentially expressed antigen of melanoma; P2RX7, P2X purinoceptor 7; TLR4, toll like receptor 4; Treg, regulatory T cell; UVC, UV light C.

Chemotherapeutics and targeted drug classes have received maximal clinical attention compared to most physical anticancer modalities baring RT and to a certain extent, PDT. However, the emergence of ICD and re-emergence of therapeutic relevance of immunotherapy has paved the way for the development of autologous or allogeneic cancer cell-based immunotherapy exploiting physical modality-induced immunogenic tumor cell death. Of note, physical anticancer modalities-based ICD might be preferable over the chemotherapeutically induced ICD for preparing cell-based immunotherapeutics since the former does not leave behind active drug residues. The main aim of this review is to discuss in detail the molecular and cell signaling properties of physical modalities inducing ICD such as RT, UVC-light, HHP, Hyp-PDT or HT. These cell death-inducing modalities are of a particular interest for designing or generating *in situ* cancer vaccines, whole cell- or DC-based vaccines for cancer immunotherapy.^53^ We discuss the evidence of ICD induced by the physical modalities in cancer patients together with a few clinical trials exploiting the whole cell or DC-based cancer vaccines using tumor cells killed by an ICD.

## Physical Modalities Inducing Tumor Immunogenicity

RT is estimated to be used as a treatment modality with curative or palliative intent in at least 50% of cancer patients.^54^ The anti-neoplastic activity of irradiation (X- or γ-rays) was believed to lie in its capacity to damage DNA and induce apoptosis of tumor cells. The abscopal effect of RT has been known for 60 y^2^ and observed in patients with various types of tumors. This suggests that RT induces ICD *in situ*^55,56^ and stimulates T cell-mediated anticancer effect. RT has been shown to induce the surface exposure of CRT^14,57^ and HSP70,^58^ and HMGB1 release.^57,59^ Irradiated tumor cells stimulate DC maturation^60^ and induce IFNγ-producing T cells *in vitro* and *in vivo*.^38,61,62^ Moreover, mice vaccinated with DCs loaded with irradiated cancer cells are immune to the challenge with live syngeneic cells.^63^ Even though RT was reported to decrease the number of T regulatory cells in some settings,^62^ regulatory T cells have been described to be more resistant to cytotoxic effect of ionizing radiation.^64^ The latter is supported by the observation that depletion of regulatory T cells potentiates the anti-neoplastic effect of RT in murine models.^54,64,65^ Interestingly, beside X- or γ-irradiation, vaccination with α-irradiated (bismuth-213) murine adenocarcinoma MC-38 also induces long-lasting protective antitumor response in mice which depends on tumor-specific T cells.^66^ MC-38 cells treated with^213−^Bi are capable of releasing DAMPs and stimulating dendritic cells *in vitro*.

The evidence for ICD induction in human cancer patients undergoing RT is scarce. Frey et al.,^67^ showed that chemotherapy treatment in combination with X-ray caused ICD in human colorectal tumor cell lines. Recently, Suzuki et al.^57^ have shown that chemoradiotherapy induces ICD in patients with esophageal squamous cell carcinoma which triggers tumor antigen-specific T cell responses. Here, HMGB1 was significantly upregulated within the tumor microenvironment and positively correlated with patient survival. As RT improves the clinical outcome of other treatment modalities such as surgery and chemotherapy, it is likely to enhance antitumor effect of various immunotherapeutic agents such as monoclonal antibodies, whole-cell or DC-based vaccines or TLR agonists.^54,68,69^ For example, a case of the abscopal effect in patient with melanoma treated with ipilimumab and RT has been reported.^71^ Current clinical studies in anticancer radioimmunotherapy have been recently extensively summarized in Vacchelli et al.^54^ Interestingly, in a recent review Frey at al.^68^ have described the combination of RT and HHP-generated whole cell tumor vaccine with the application of IL-12 in an immunotherapeutic protocol. Similarly, Sipuleucel-T®, the currently only FDA-approved antigen presenting cell-based cancer vaccine for the treatment of asymptomatic metastatic castration resistant prostate cancer^71^ is being evaluated in clinical trial together with RT^54^ (**Table 2**).

**Table 2.** The list of clinical trials which involve the preparation of tumor cells killed by an ICD-inducing physical modality for the use as whole cell- or DC-based vaccines in cancer immunotherapy

Indications	Status	Phase	Type of physical modality applied	Notes	Ref.
Prostate cancer	Recruiting	II	RT	castrate resistant metastatic prostate cancer (CRPC)	NCT01807065
Prostate cancer	Recruiting	II	HHP	After radical primary prostatectomy	NCT02107404
Prostate cancer	Ongoing, recruited	II	HHP	After primary radiotherapy, with high risk	NCT02107430
Prostate cancer	Ongoing, recruited	II	HHP	CRPC combined with docetaxel chemotherapy	NCT02105675
Prostate cancer	Ongoing, recruited	II	HHP	CRPC combined with hormone therapy	NCT02107391
Prostate cancer	Recruiting	II	HHP	2nd treatment cycle of vaccine in localized cancer	NCT02137746
Prostate cancer	Recruiting	III	HHP	CRPC combined with docetaxel chemotherapy	NCT02111577
Ovarian cancer	Recruiting	II	HHP	Newly diagnosed patients with chemotherapy	NCT02107937
Ovarian cancer	Recruiting	II	HHP	Relapsed platinum resistant ovarian cancer	NCT02107378
Ovarian cancer	Recruiting	II	HHP	Relapsed gemcitabine resistant ovarian cancer	NCT02107950
HNSCC	Trial to be open by 2017	I	PDT	As adjuvant to surgery for advanced HNSCC	Personal Communication (SO Gollnick, Roswell Park Cancer Institute, USA)

Abbreviations: CRPC, castrate resistant metastatic prostate cancer; DC, dendritic cells; HHP, high hydrostatic pressure; HNSCC, Head and Neck squamous cell carcinoma; PDT, photodynamic therapy; RT, radiotherapy;

### Ultraviolet C light

Ultraviolet light (UV) refers to electromagnetic radiation with a wavelength shorter than visible violet light but longer than X- and γ-rays. According to the wavelength range, UV light can be divided into UVA (400–320 nm), UVB (320–280 nm) and UVC (280–200 nm).^72^ In cells UV light affects mainly DNA which leads to the apoptosis or necrosis depending on the cell type.^14,59,72^ However, only UVC-light treatment at 10–120 nm, technically with properties of ionizing radiation, was shown to induce ICD in tumor cells which was accompanied by the pre-apoptotic exposure of CRT^14^ on the cell surface and HSP70 and HMGB1 release into the cell culture medium at later time points.^59^ Various molecular determinants and pathways of UVC-light-mediated ICD await further elucidation. The ability of UVC but not UVA or UVB light to induce ICD has been an enigma which remains unexplored. UVA, UVB, and UVC are all capable of stimulating ROS production^73^ as well as overlapping stress response pathways^74^ including ER stress.^75^ Which particular subtle difference distinguishes between ICD and non-ICD is an avenue worth investigating.

UVC-light has been known for more than 30 y to induce an inflammatory response in skin.^76^ In 1991 Begovic et al.^77^ showed that vaccination of immunocompetent mice with UVC-irradiated tumor cells conferred immunity to subsequent re-challenge with live tumor cells in contrast to immunodeficient mice which developed tumors. This tumor-growth inhibiting effect was mediated by CD8^+^ T cells and NK cells. UVC-treated tumor cells were shown to stimulate phagocytosis and DC maturation which in turn lead to the stimulation of IFNγ producing CD8^+^ T cells.^59^ Moreover, DCs stimulated with UVC-treated cancer cells upregulated genes connected to antigen processing and proinflammatory cytokines.^38,59^ The effect of UVC irradiation on tumor cells has recently been tested in a model of superficial brain cancer and metastasis.^78^ UVC irradiation, beamed through the craniotomy open window, induced apoptosis in tumor cells which led to a significantly extended survival of experimental animals. In humans, there are no clinical studies involving UVC treatment of tumors, possibly due to a high pro-tumorigenic mutation rate induced by UVC light.^38^

### High hydrostatic pressure

HHP between 1 and 100 megapascal (MPa) is considered to be physiological, and it induces reversible morphological changes and a mild stress response. HHP between 100–150 MPa induces apoptosis of murine cells, HHP between 150–250 MPa affects the viability of human cells, whereas HHP treatment between 300–400 MPa (dependant of the cell type) leads to cell necrosis.^79-81^ HHP treatment causes cell rounding, cytoplasmic gelification, the inhibition of enzymatic functions and synthesis of cellular proteins. However, DNA does not seem to be affected by HHP below 1000 MPa.^81^ In biotechnology, HHP is applied to sterilize food, human transplants and pharmaceuticals.^81,82^ The use of HHP as a cancer treatment modality was described in 1972 for the treatment of bladder carcinoma.^83^ The hydrostatic bladder dilatation method was subsequently tested in cancer patients in a small clinical trial.^84^ Later the group of Shinitzky showed that vaccination by HHP-killed tumors cells treated with a chemical crosslinker adenosine dialdehyde alone and in combination with a reducing agent N-acetyl-L-cysteine induced antitumor immunity in mice^85,86^ and exhibited immunogenicity *in vitro*.^87,88^ The cell death induced by HHP was investigated in greater detail by the group of Udo Gaipl who suggested that HHP might be a promising technique for a generation of whole cell-based anticancer vaccines.^79-81^ Apoptotic cells treated with HHP were shown to release HSP70 and HMGB1 and possess immunogenicity *in vivo* which was determined by tumor-specific antibodies.^49,80^ Recently, Fucikova et al.^89^ have shown that HHP is a potent inducer of ICD of human prostate and ovarian cancer cell lines as well as in acute lymphocytic leukemia cells which leads to the exposure of CRT, HSP70 and HSP90 molecules on the cell surface and the release of HMGB1 and ATP from the dying cells. More importantly, DCs loaded with HHP-killed tumor cells displayed an enhanced phagocytic capacity, expressed high levels of co-stimulatory molecules, and stimulated high numbers of tumor-specific T lymphocytes without inducing T regulatory cells in the absence of any additional immunostimulants.^89^ HHP-induced tumor cell death was shown to fulfill all currently described molecular criteria of ICD, including the activation of analogous intracellular signaling pathways similar to anthracyclines^15^ and Hyp-PDT (see below).^26^ Accordingly, an increased production of ROS, phosphorylation of eIF2α, the activation of caspase-8 and caspase-8-mediated cleavage of BAP31 was detected.^89^ The immunogenicity of HHP-killed tumor cells *in vivo* is currently being evaluated in therapeutic as well as prophylactic settings in mouse cancer models.

**Figure 2.**
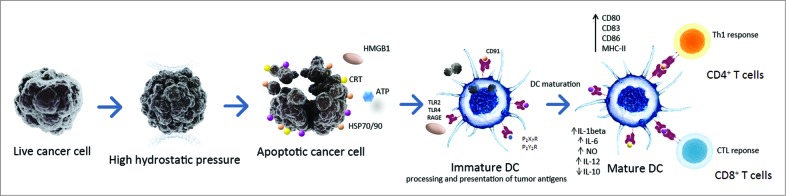
A schematic representation of DC-based vaccine preparation using immunogenic HHP-killed tumor cells. Tumor cells treated with HHP (or other physical ICD-inducing modalities) expose various danger signals, so called DAMPs, in different stages of apoptosis. These DAMPs include calreticulin (CRT), heat shock proteins 70/90 (HSP70/90), HMGB1 and ATP. These molecules bind to respective cognate receptors like CD91 (for CRT/HSPs), TLR2/TLR4 (for HMGB1 or HSP70), P2RX7/P2RY2 (for ATP), respectively, on the cell surface of DCs. This leads to an enhanced engulfment of tumor cells and DC maturation characterized by upregulation of costimulatory molecules such as CD80, CD83, CD86 and HLA-DR, and by a distinct pro-inflammatory cytokine pattern. Activated DCs efficiently present tumor-specific antigens in the context of MHC class I and II molecules to T cells, inducing antitumor CD4^+^ and CD8^+^ T cell responses. Abbreviations: ATP, Adenosine triphosphate; CRT, calreticulin; CTL, cytotoxic T lymphocytes; DAMPs, danger-associated molecular patterns; DC, dendritic cell; HHP, high hydrostatic pressure; HMGB1, high mobility group box 1 HSP70, heat shock protein 70; HSP90, heat shock protein 90; ICD, immunogenic cell death; P2RX7, P2X purinoceptor 7; P2RY2, P2Y purinoceptor 2; RAGE, receptor for advanced glycation endproducts; TLR, toll like receptor.

HHP treatment of tumor cells can be easily standardized and performed in GMP conditions to allow its incorporation into manufacturing protocols for cancer DC-based immunotherapy product. Multiple clinical trials for prostate and ovarian cancer^90^ have now been initiated to evaluate the potential of DC-based cancer vaccine loaded with HHP-treated cancer cells to induce tumor cell-specific immune responses and modify the clinical course of the disease (**Table 2**). A schematic representation of DC-based vaccine preparation using immunogenic HHP-treated tumor cells which could be applied to other physical tumor cell death-inducing modalities is shown in **Fig. 2**.

### Photodynamic therapy

Over the last decades, PDT has been explored as a promising anticancer treatment due to its relative specificity as well as the absence of harmful side effects usually associated with chemotherapy and RT. PDT has a two-step modus operandi involving administration of mainly tumor-localizing photosensitizer followed by its activation with a light of specific wavelength, which ultimately leads to the photochemical production of ROS, thereby causing oxidative stress-based cell death. The most attractive attribute of PDT is that, this oxidative stress can be directed toward a particular subcellular organelle or locale within the cancer cells, due to the tendency of a given photosensitiser to exhibit a certain degree of “tropism” toward a particular subcellular site e.g., Hypericin tends to be reticulotropic since it mainly localizes in the ER membrane.^91^ The extent of PDT-induced damage is multifactorial depending on, among others, the type of tumor cell, photosensitizer type and its subcellular localization, and the cellular oxygen levels as well as light irradiation fluency. PDT-induced antitumor effects include cytotoxicity toward tumor cells, tumor-infiltrating cells and vasculature as well as the activation of the complement cascade and recruitment of immune cells like DCs or neutrophils to the tumor site.^92,93^ The molecular mechanisms of PDT-mediated cell death depend strongly on the subcellular localization of the photosensitizer and the PDT dosage.^94^ At high fluence PDT in general tends to induce necrosis while high to medium fluence induce either a mixture of apoptosis and necrosis or predominantly apoptosis in a dose-dependent fashion.^91^

It is noteworthy though, that due to the essential involvement of a chemical component, namely a photosensitizer/pro-drug, PDT unlike RT or HHP, cannot be considered as an exclusively physical modality but rather a physicochemical anticancer modality. However, since in the absence of the physical component (i.e., specific wavelength of light used for activating a particular photosensitizer), PDT would be unable to exert its bona fide anticancer effects (i.e., high cancer cell death, ICD or antitumor immunity in general),^26,93^ we have included a discussion of the relevant immunogenic features of this physicochemical anticancer modality along with the actual physical procedures, like RT and HHP.

Since early work in 1970s, there have been over 200 clinical trials involving PDT alone or in combination with other treatment modalities of various cancers.^93^ However, very little is known about the impact of PDT on the human immune system. It has been shown that local tumor PDT can enhance systemic antigen-specific immune responses against tumors in patients^95^ and can also induce clinical abscopal effect-like immune response against distant non-treated tumors.^96^ Importantly, Garg et al.^26,51^ have recently shown that specifically Hyp-PDT induces ICD in murine and human systems. Hyp-PDT is the first Type II ICD inducer to be characterized, and it is by far the most effective inducer of ROS-based ER stress among all the known ICD inducers.^51^ Since Hypericin localizes prevalently in the ER, its light-activation causes ROS-based ER stress that culminates into mitochondrial apoptosis.^97^ Hyp-PDT has been observed to induce signatures of ER stress in a treated bladder carcinoma tumor *in vivo*.^98^ Moreover, Hyp-PDT has been applied in clinical trials with some success for the treatment of patients with non-melanoma skin cancer,^99^ cutaneous T-cell lymphoma^100^ mesothelioma^101^ and basal or squamous cell carcinoma.^102^

Hyp-PDT induces all the major molecular and immunological hallmarks of ICD. Uniquely, Hyp-PDT induces pre-apoptotic active emission of four crucial DAMPs i.e., surface exposed CRT, surface exposed HSP70, surface exposed HSP90 and secreted ATP^26,51,103^ (Dudek et al. unpublished results). This is followed by passive, late apoptotic, release of chaperokines like HSP70/HSP90. Hyp-PDT-treated cancer cells are preferentially phagocytosed in a surface CRT-dependent fashion by various innate immune cells including murine and human DCs which undergo efficient phenotypic and functional maturation.^26^ These fully mature DCs thereafter induce efficient proliferation and clonal expansion of human IFNγ-producing CD4^+^ and CD8^+^ T lymphocytes^104^ – an important sign of activation of anticancer immune effector mechanisms. In line with this, Hyp-PDT elicited ICD has been found to be capable of mediating efficient tumor rejection *in vivo* in murine prophylactic as well as therapeutic/curative vaccination models.^16,91^

PDT, in general, has been shown to be suitable for vaccine generation, as immunization with PDT-killed tumor cells or cell lysate induces strong antitumor immunity in mice^105,106^ also in the absence of any adjuvants.^107^ In addition, photoimmunotherapy with DCs loaded with PDT-treated tumor cells has been shown to stimulate the cytotoxicity of T and NK cells toward tumors in mice^108^ suggesting its clinical potential. However, despite some clinical success in using PDT in cancer treatment, there are no clinical data on the use of PDT-based cancer vaccines in immunotherapy. There is currently a clinical trial in preparation for application of PDT-based vaccines (**Table 2**). Of note, Hyp-PDT-induced ICD based DC vaccines are currently being tested in preclinical trials for glioblastoma and ovarian cancer (Garg et al. unpublished data; Immunotherapy Platform Leuven or ITPL, UZLeuven, Belgium). The success of such preclinical trials and preclinical optimizations would define the possible clinical translation of PDT-based immunotherapy in the near future.

### Hyperthermia

HT refers to the administration of heat locally as well as systemically (whole-body HT). Since the 1970s numerous pre-clinical studies on the effects of heat on tumor cells have been performed *in vitro* as well as in experimental animal models. In clinical oncology, HT has been shown to be a potent sensitizer for the conventional chemo- or RT-treatment and improved patient's survival in various clinical trials.^109^ Clinical HT was shown to affect innate and adaptive immunity; stimulating antigen presentation, maturation and migration of DCs, as well as homing of T lymphocytes to lymph nodes thereby facilitating T cell priming. The efficiency of heat-induced killing of tumor cells depends mainly on the applied temperature (ranging in most studies from 41°C to 44°C), the duration of heat treatment, tumor cell type and the cell cycle phase. Malignant cells are capable of thermotolerance induced by heat-shock response which is accompanied by the expression of HSPs and other post-translational adaptation processes.^109^ HT was shown to cause apoptosis mainly at lower temperatures (41–43°C) (and predominantly necrosis at higher temperatures (>43°C).^110^ Increased immunogenicity of mouse and human tumor cells subjected to HT as well as the induction of tumor-antigen specific T cell responses *in vitro* and *in vivo* has been well documented.^111-113^ The major technical problem with HT application is the difficulty to heat specifically only the tumor region without inducing damage to the normal tissue. In recent years, the development of new techniques based on magnetic nanoparticles^114^ allowed the induction of such tumor-specific HT. The group of Kobayashi has developed a HT system based on liposomes containing magnetic nanoparticles which caused necrotic tumor cell death and the release of HSP70, thereby stimulating an antitumor immune response *in vivo*.^115,116^ Recently, magnet-mediated HT at high therapeutic temperatures (50–55°C) was shown to induce abscopal antitumor immune effects on Walker-256 carcinosarcomas in rats.^117^

Currently, it is unclear, whether HT treatment alone can induce bona fide ICD. HT-treatment (<43°C) was shown to induce ER stress.^118^ Also HMGB1 release was detected after HT treatment of tumor cells lines at high temperature of 56°C.^59^ The current paradigm of the immunogenicity of HT lies in the action of HSP70 and/or other released heat shock proteins which via TLR4 signaling play the main role in the initiation of tumor-specific immune responses.^111,112,119,120^ It has been shown that the combination of HT and RT (X-rays or UVC) induces an inflammatory necrotic tumor death which can be monitored by the release of HMGB1 and HSP70^121,122^ and stimulation of DC maturation and release of pro-inflammatory cytokines.^59,123^ Currently there are no clinical data on the use of HT-killed tumor cells alone in clinical protocols.

## Conclusion

Malignant diseases represent a major challenge in human medicine. Combined therapeutic regimens like surgery, RT and chemotherapy can efficiently reduce the tumor volume and render cancer cells visible for immune attack, thereby improving the prognosis of cancer patients. However, the occurrence of metastatic disease and the reservoir of cancer stem cells still remain the greatest challenge in combating cancer. Therefore the combination of the cancer cell killing with the concomitant help of the host immune system to mount a competent anticancer immune response is an attractive therapeutic aim. Physical cell death-inducing modalities like PDT or HT have been proven to be able to act as *in situ* vaccines, and to aid in inducing antitumor immunity in human patients; PDT most likely via ICD induction. Moreover, these modalities, especially HHP which is a potent inducer of ICD in tumor cells, might have a great potential in the development of new whole cell-based or DC-based vaccines. More research is however needed on the molecular mechanism of ICD induction by PDT, HHP and possibly by HT, as well as by the currently used chemotherapeutic agents and irradiation to optimize the therapeutic approaches. Efforts should also be made to incorporate the design of new modern immunotherapeutic strategies based on ICD inducers into current multimodal therapeutic protocols.
